# Factors influencing young people’s transitions from child to adult mental health services in Wales

**DOI:** 10.23889/ijpds.v9i5.2484

**Published:** 2024-09-18

**Authors:** Louisa Roberts, Sophie Wood, David Wilkins, Katherine Shelton

**Affiliations:** 1 Cardiff University; 2 CASCADE

## Objectives

This presentation provides an overview of young peoples’ transitions from child to adult mental health services in Wales. Transitions are unstable times where need for support can be greatest, yet many young adults fail to qualify for ongoing specialist support. Young people with social care needs and/or living in poverty face an increased likelihood of mental ill health. These underlying factors of disadvantage are modifiable and can be considered ‘solution linked variables’ (O’Campo and Dunn, 2011) of particular relevance in public health policy development.

## Approach

This study positions intersectionality theory within a quantitative, secondary data methodology. Descriptive statistics and logistic regression models were used to elicit how social care need, deprivation, mental health diagnoses, gender and ethnicity might interact to influence likelihood of transition from child to adult mental health services. Sequence analyses developed pathway typologies from varying patterns of mental health service access. 

## Results

Novel findings reflecting the interactional influences of differing social care need and area levels of deprivation on child to adult mental health service transitions will be presented alongside results controlling for gender, ethnicity and mental health diagnoses. The value of mapping typologies of mental health service use will be discussed in relation to the findings.

## Conclusions

Interim findings suggest greatly increased mental health service use and differing patterns of access for young people with social care needs and/or living in the most deprived areas as they transition to adulthood. The implications for service development and delivery in times of unprecedented demand are explored.

